# Association between C-reactive protein and coronary calcium score in coronary artery disease

**Published:** 2009-04

**Authors:** Ali Hosseinsabet, Ahmad Mohebbi, Alireza Almasi

**Affiliations:** Department of Cardiology, Shaheed Rajaei Cardiovascular Center, Iran University of Medical Sciences, Tehran; Department of Cardiology, Shaheed Rajaei Cardiovascular Center, Iran University of Medical Sciences, Tehran; Department of Radiology, Shaheed Rajaei Cardiovascular Center, Iran University of Medical Sciences, Tehran

## Abstract

**Background:**

Both high-sensitivity C-reactive protein (hs-CRP) and spiral computed tomography coronary artery calcium score (CCS) are valid markers of cardiovascular risk. It is unknown whether hs-CRP is a marker of atherosclerotic burden or if it reflects a process leading to acute coronary events.

**Methods and results:**

We studied the association between hs-CRP and CCS in 143 patients who were candidates for coronary artery bypass grafting (CABG). In our cross-sectional study, we found no significant association between hs-CRP and the CCS in bivariant (*p* = 0.162) and multivariant (*p* = 0.062) analyses. However, in patients who did not use statins, this association was significant and positive in the bivariant analysis (*p* = 0.001), but in the multivariant analysis it was negative and significant (*p* = 0.008).

**Conclusion:**

High-sensitivity CRP was not correlated with CCS. The relationship between CRP and clinical events might not be related to atherosclerotic burden. Measures of inflammation, such as hs-CRP, and indices of atherosclerosis, such as CCS, are likely to provide distinct information regarding cardiovascular risk.

## Summary

Much evidence suggests that inflammation plays a major role in the development of atherosclerosis and its clinical manifestations. 1,2 In some studies, plasma levels of inflammatory markers, particularly high-sensitivity C-reactive protein (hs-CRP), predicted myocardial infarction and cardiovascular death.^3^-^8^ However, hs-CRP is associated with many established risk factors, including dyslipidaemia, cigarette smoking, hypertension, diabetes and obesity.^9^-^15^ The relationship between hs-CRP and coronary artery disease (CAD) was found to be positive and significant in somestudies,^16^-^18^ but not in others,^17^,^19^-^27^ and negatively significant in yet others.^28^,^29^ The extent to which hs-CRP levels predict clinical events depends on the relationship of hs-CRP to the burden of underlying atherosclerosis or the milieu leading to plaque rupture and thrombosis, which is unknown at present. Given that hs-CRP levels predict clinical events, it is of substantial interest to dissect the pathophysiology of this relationship.

In contrast to clinical events, an independent association between hs-CRP levels and coronary^19^-^29^ or carotid^27^,^30^-^36^ atherosclerosis has not been clearly established. Coronary artery calcification (CAC), measured by electron beam tomography (EBT) or spiral computed tomography might be useful in identifying novel risk factors for coronary atherosclerosis in asymptomatic subjects. The amount of CAC found with EBT is correlated with the burden of atherosclerosis found both with coronary angiography and at autopsy,^37^,^38^ and studies suggest that CAC is a predictor of clinical CAD events in both symptomatic^39^ and asymptomatic^40^,^41^ subjects. Studies of CAC might permit differentiation of factors associated with coronary atherosclerosis from those related to plaque rupture or thrombosis.

Studies of hs-CRP and CAC in healthy subjects have produced conflicting results. Whereas some found no association between hs-CRP and CAC,^17^-^29^ others have reported a weak relationship.^16^-^18^ It is unclear whether these conflicting reports reflect the limitations of the study design and analysis or real differences in the pathophysiology of CAC, a measure of coronary atherosclerotic burden, and elevated hs-CRP, a marker of inflammation. Some support this concept that coronary calcium scores (CCS) and plasma hs-CRP levels may provide independent and complementary information regarding the risk of cardiovascular events.^22^,^42^

## Materials and methods

The study population consisted of 143 patients with coronary artery disease who were admitted to Shaheed Rajaei Cardiovascular Center, a tertiary academic referral centre, for coronary artery bypass grafting (CABG) between December 2006 and March 2007. When patients were admitted to our centre for CABGs, history taking and physical examinations were done. Exclusion criteria were: a history of myocardial infarction or unstable angina in the previous month; a history of prior aortic valve replacement or mitral valve replacement; and a prior history of CABG or coronary stenting.

All study participants gave written informed consent. The protocol was approved by the Research Committee at the Iran University of Medical Sciences, Tehran. Age, and cardiac risk factors including hypertension, dyslipidaema, diabetes mellitus, family history of coronary disease, smoking status and drug history were determined by interview (self-reported), and body mass index (BMI) was measured.

Blood sampling was done for lipid profiles and creatinine^43^-^45^ and hs-CRP levels, and blood samples were frozen at –70°C for four months. A single laboratory technician, blinded to all clinical and radiological data, carried out the test for hs-CRP, using the latex immunoturbidity assay (detection limit 0.1–10 mg/l and coefficient of variation 1%) from commercial kits (Pars Azmun Co).

Coronary calcium scoring was done by 10-slice spiral CT scan (Siemens Somatom Sensation 10). Calcium scores of the coronary artery were expressed according to Agatston and colleagues.^46^ A total CAC score was determined from the sum of individual scores of the four major epicardial coronary arteries. A single radiologist blinded to all clinical and serological data interpreted all scans.

## Statistical analysis

Data were analysed by SPSS 15 software and reported as means ± SD if they were continuous, and as proportions if they were categorical. Because some variables did not have normal distributions, we transformed them logarithmically for normalisation of data. Some patients had a CCS of 0, so log (CCS + 1) was substituted.

Firstly, we assessed the overall association between log (CCS + 1) and log (hs-CRP) using the Pearson correlation coefficient, and then included age, gender, risk factors and any drugs used. Because almost all patients used aspirin and beta-blockers and very few used calcium channel blockers or gemfibrozil, we did not enter these variables in our analysis. Secondly, we assessed this correlation with multivariate linear regressions (enter mode) overall and then according to statin usage. We entered age, BMI, drug history, all risk factors, lipid profile and creatinine levels in the multivariate analysis.

## Results

Table 1 shows demographic characteristics, hs-CRP levels and CCS scores in the sample (*n* = 143). Bivariant analysis of the correlation between hs-CRP and CCS in all patients and subgroups showed it was not significant overall (*r* = – 0.118, *p* = 0.162), but was significant in the 60- to 69-year-old patients (*r* = 0.327, *p* = 0.031) and in patients not using statins (*r* = 0.442, *p* = 0.001). These correlations were moderate and significant. In other subgroups, this correlation was not significant. Factors associated with CCS when hs-CRP was not included in the adjusted multivariate linear regressions are shown in Table 2. Age, male gender and family history of coronary artery disease were positive predictors of CCS.

**Table 1:** Characteristics Of The Study Sample

Age (years)	57.7 ± 9.4
< 50	18.2
50–59	39.2
60–69	30.8
> 70	11.9
BMI (kg/m^2^)	27.2 ± 3.5
< 24.99	29.4
25–29.99	49
> 30	21.6
TG (mmol/l)	1.73 ± 0.88
Cholesterol (mmol/l)	4.46 ± 1.27
LDL (mmol/l)	4.43 ± 0.81
HDL (mmol/l)	1.06 ± 0.98
CR (mmol/l)	121.1 ± 84
hs-CRP (mg/l)	2.89 ± 3.43
CCS	366.4 ± 586.7
Male	74.1
HTN	32.2
DLP	45.5
DM	32.9
C/S	35
FH	14
ACEI/ARB	51.7
Statins	62.2

Values are mean ± SD, or percentage. BMI = body mass index; TG = triglycerides; LDL = low-density lipoprotein; HDL = high-density lipoprotein; CR = creatinine; hs-CRP = high-sensitivity CRP; CCS = coronary calcium score; HTN = hypertension; DLP = dyslipidaemia; DM = diabetes mellitus; C/S = cigarette smoking; FH = family history of coronary artery disease; ACEI/ARB = angiotensin converting enzyme inhibitors/angiotensin receptor blockers.

**Table 2:** Multivariate Analysis Of Factors Associated With Ccs When hs-CRP Was Excluded From Analysis

	*B*	*SD*	*p*
(Constant)	1.173	1.323	0.377
Age (years)	0.034	0.008	0.000
Gender	–0.409	0.191	0.035
HTN	0.304	0.177	0.089
DLP	0.019	0.163	0.909
DM	0.121	0.165	0.464
FH	0.470	0.212	0.028
C/S	0.058	0.172	0.735
ACEI/ARB	–0.069	0.153	0.651
Statins	–0.146	0.157	0.355
LDL	0.000	0.003	0.859
Log HDL	0.138	0.184	0.455
Log TG	–0.182	0.159	0.257
Log CR	–0.134	0.252	0.598
BMI	–0.014	0.021	0.514

Results of linear regression [log of (CCS + 1) as the dependent variable] are presented when log hs-CRP is not included in the analysis, as the change log (CCS + 1) for a specific change in risk factor. Model was adjusted for the following variables; age, gender, history of hypertension, history of dyslipidaemia, diabetes mellitus, family history of coronary artery disease, smoking, use of the following medications: statins, ACEI/ARB. LDL [log LDL], HDL [log HDL], TG [log TG], CR [log CR], body mass index.

Factors associated with CCS when hs-CRP was included in the adjusted multivariate linear regressions are shown in Table 3. Age was the only predictor of CCS in the presence of hs-CRP, and gender and family history of coronary artery disease were not predictors of CCS after adjustment for hs-CRP levels. Because in bivariant analysis the association of hs-CRP and CCS was significant in patients who did not use statins, we analysed this association in these patients in adjusted multivariate linear regressions. Table 4 shows this analysis. Male gender and family history of coronary artery disease were positive predictors of CCS, and hs-CRP was a negative predictor of CCS (*p* = 0.008) in patients who did not use statins.

**Table 3:** Multivariate Analysis Of Factors Associated With Ccs When hs-CRP Was Included In Analysis

	*B*	*SD*	*p*
(Constant)	1.046	1.312	0.427
Age (years)	0.037	0.008	0.000
Gender	–0.343	0.193	0.078
HTN	0.293	0.176	0.099
DLP	–0.005	0.161	0.977
DM	0.141	0.164	0.392
FH	0.395	0.213	0.067
C/S	0.068	0.170	0.688
ACEI/ARB	–0.032	0.153	0.834
Statins	–0.204	0.158	0.200
LDL	0.001	0.003	0.657
Log HDL	0.089	0.184	0.630
Log TG	–0.169	0.158	0.288
Log CR	–0.063	0.253	0.802
BMI	–0.013	0.021	0.542
Log hs-CRP	–0.115	0.061	0.062

Results of linear regression [log of (CCS + 1) as the dependent variable] are presented when log hs-CRP is included in analysis as the change log (CCS + 1) for a specific change in risk factor. Model was adjusted for the following variables; age, gender, history of hypertension, history of dyslipidaemia, diabetes mellitus, family history of coronary artery disease, smoking, use of the following medications: statins, ACEI/ARB. LDL [log LDL], HDL [log HDL], TG [log TG], CR [log CR], body mass index and hs-CRP [log hs-CRP].

**Table 4:** Multivariate Analysis Of Factors Associated With CCS In Patients Not Using Statins

	*B*	*SD*	*p*
(Constant)	3.774	1.682	0.031
Age (years)	0.021	0.012	0.088
Gender	–0.653	0.262	0.017
HTN	0.318	0.259	0.227
DLP	0.086	0.243	0.724
DM	0.250	0.226	0.276
FH	0.682	0.318	0.038
C/S	–0.346	0.275	0.215
ACEI/ARB	0.191	0.231	0.414
LDL	0.004	0.004	0.294
Log HDL	0.188	0.219	0.396
Log TG	–0.261	0.241	0.285
Log CR	–0.531	0.292	0.077
BMI	–0.068	0.037	0.077
Log hs-CRP	–0.278	0.100	0.008

Results of linear regression [log of (CCS + 1) as the dependent variable] are presented in patients who not use statins, as the change log (CCS + 1) for a specific change in risk factor. Model was adjusted for the following variables; age, gender, history of hypertension, history of dyslipidaemia, diabetes mellitus, family history of coronary artery disease, smoking, use of the following medications: statins, ACEI/ARB. LDL [log LDL], HDL [log HDL], TG [log TG], CR [log CR], body mass index and hs-CRP [log hs-CRP].

## Discussion

Traditionally, the risk of a clinical coronary event reflects the burden of underlying coronary atherosclerosis, factors that lead to plaque rupture and those that promote thrombus formation. Histopathological studies have proved that coronary artery calcification is strongly associated with total plaque burden. The amount of CAC measured with EBT was correlated with the burden of atherosclerosis both with coronary angiography and at autopsy,^37^-^38^ and preliminary studies suggest that CAC is a predictor of clinical CAD events in both symptomatic^39^ and asymptomatic subjects.^40^-^41^ Studies of CAC might permit differentiation of factors associated with coronary atherosclerosis from those related to plaque rupture or thrombosis. CCS, measured with spiral CT, might be useful for exploring the relationship of risk factors with coronary atherosclerosis.

We examined the association between plasma hs-CRP and CCS in patients who were candidates for CABGs. In previous studies, subjects of these studies were suspected to have coronary artery disease but without any evidence. In our study, we selected patients who had coronary artery disease documented by selective coronary artery angiography. We found no evidence of a positive association between hs-CRP and calcium scores. Indeed, if anything, these data suggested an inverse relationship between hs-CRP levels and coronary calcium in patients who did not use statins. Nonetheless, we believe the lack of a positive association between hs-CRP and coronary calcium score deserves careful consideration.

The lack of correlation in the current data between spiral CT coronary calcium score and hs-CRP suggests that calcification may be less likely to reflect inflammation *per se*. Spiral CT-detected calcification may predominantly be a marker for mature and hence stable atherosclerotic plaque, and therefore only be an indirect marker for the presence of uncalcified rupture-prone lesions, which may be more likely markers for future cardiac events. However, correlation between soft, non-calcified plaque and hs-CRP has not been confirmed.^24^

The deposition of calcium in atherosclerotic lesions has been shown to be an active process analogous to the formation of bone spicules.^47^ Therefore coronary calcification may not merely be a direct consequence of atherogenesis but rather may depend on the presence of specific determinants independent of the central processes involved in plaque formation. Our finding supports the concept that the hs-CRP level might not be related to atherosclerosis, but it may be a marker of plaque rupture and thrombosis. Therefore, hs-CRP might not be useful in identifying the underlying mechanisms of the initiation or progression of atherosclerosis.

We used a validated commercial assay for the measurement of hs-CRP, but variability in commercial assays may limit the validity of these data. We used CCS as a surrogate for coronary atherosclerotic plaque burden on the basis of the well-established relationship between CCS and the extent of histological plaque.^37^ However, atherosclerosis in vascular beds other than the coronary arteries could also contribute to the level of hs-CRP.^48^

## Conclusion

This study demonstrated that hs-CRP is unrelated to the presence and severity of clinical calcified atherosclerosis. It suggests that serological inflammatory markers are principally a measure of the athero-inflammatory disease process and are not an index of the extent of coronary atherosclerotic plaque. Because CCS and hs-CRP are associated with risk for subsequent cardiovascular events, these two measures may be complementary rather than competitive for risk prediction.
